# Supplementary material for: Archaeal Signalling Networks—New Insights Into the Structure and Function of Histidine Kinases and Response Regulators of the Methanogenic Archaeon Methanosarcina acetivorans
Source: Environ Microbiol. 2025 Jan 31;27(2):e70047. doi: 10.1111/1462-2920.70047 (PMC11784639; doi:10.1111/1462-2920.70047)
Supplement: Supplementary file 1 — Data S1: Supporting Information. [file EMI-27-e70047-s001.pdf]

Supplementary Material

Archaeal signalling networks - new insights into the structure and function of histidine kinases and response regulators of the methanogenic archaeon *Methanosarcina acetivorans*

Nora FK Georgiev [1], Anne L Andersson [1], Zoe Ruppe [1], Loriana Kattwinkel [1] and Nicole Frankenberg-Dinkel [1,*]

[1]Rheinland-Pfälzische Technische Universität Kaiserslautern-Landau, Department of Microbiology

[*]Corresponding author: nicole.frankenberg@rptu.de

Table S1: Microbial strains

Strain	Genotype	Reference
E. coli strains		
E. coli DH5α	F^- *endA1 glnV44 thi-1 recA1 relA1 gyrA96 deoR nupG purB20* φ80d*lacZ*ΔM15 Δ(*lacZYA-argF*)U169, *hsd*R17(r_K^- m_K^+), λ^-	(Hanahan, 1983)
E. coli Bl21 (DE3)	F^- *ompT gal dcm lon hsdS*$_B$(r_B^- m_B^-) λ(DE3 [*lacI lacUV5-T7p07 ind1 sam7 nin5*]) [*malB*$^+$]$_{K-12}$(λ^S)	(Studier & Moffatt, 1986)
E. coli Nissle 1917	serotype O6:K5:H1	(Grozdanov *et al.*, 2004)
E. coli C43 (DE3)	F^- *omp*T *hsdS*B(r_B^- m_B^-) *gal dcm* (DE3)	(Miroux & Walker, 1996)
E. coli TKR 2000	*ΔkdpFABCDE trkA405 trkD1 atp706*	(Kollmann & Altendorf, 1993)
M. acetivorans strains		
M. acetivorans WWM73	Δ*hpt*::P*mcrB*-*tetR*-ϕC31-*int*-attP	(Guss *et al.*, 2008)

Table S2: Vectors and plasmids used in this study

Plasmid	Characteristic	Reference
pASK-IBA3	Expression vector, heterologous overexpression in *E. coli*, Strep-tag II, *tet* promotor, Amp^R	IBA Lifescience GmbH
pACYCDuet-1	Expression vector, heterologous overexpression and coexpression in *E. coli*, His-tag and S-tag, T7 promoter, Cm^R	Novogene Co, Ltd.
pET21a(+)	Expression vector, heterologous overexpression in *E. coli*, T7-tag and His-tag, T7 promotor, Amp^R	Novogene Co, Ltd.
pASK-IBA3-MA_4377	pASK-IBA3 derivate, coding region of MA_4377 from *M. acetivorans* at *Sac*II/*Nco*I with C-terminal Strep-tag II, *tet* promoter, Amp^R	(Sexauer, 2021)
pASK-IBA3-MA_4377-CHPK	pASK-IBA3 derivate, coding region of MA_4377 (CHASE-HK domain) from *M. acetivorans* at *Bam*HI/*Nco*I with C-terminal Strep-tag II, *tet* promoter, Amp^R	This study
pASK-IBA3-MA_4377-PKR1R2	pASK-IBA3 derivate, coding region of truncated cytosolic MA_4377 (HK-R2 domain) from *M. acetivorans* with C-terminal Strep-tag II, *tet* promoter, Amp^R	(Sexauer, 2021)
pASK-IBA3-MA_4377-$PK_{H497Q}R1_{D818N}R2$	pASK-IBA3-MA_4377-PKR1R2 derivate with amino acid exchange: His497 to Gln and Asp818 to Asn, obtained by site-directed mutagenesis	This study
pASK-IBA3-MA_4377-PKR1	pASK-IBA3 derivate, coding region of truncated cytosolic MA_4377 (HK-R1 domain) from *M. acetivorans* at *Bam*HI/*Nco*I with C-terminal Strep-tag II, *tet* promoter, Amp^R	This study
pASK-IBA3-MA4_377-$PK_{H497Q}R1$	pASK-IBA3-MA4_377-PKR1 derivate with amino acid exchange: His497 to Gln, synthetic gene	This study
pASK-IBA3-MA4_377-PK	pASK-IBA3 derivate, coding region of truncated cytosolic MA_4377 (only HK domain) from *M. acetivorans* with C-terminal Strep-tag II, *tet* promoter, Amp^R	(Sexauer, 2021)
pASK-IBA3-MA4_377-PK_{H497Q}	pASK-IBA3-MA_4377-PK derivate with amino acid exchange: His497 to Gln, Synthetic gene	This study

pASK-IBA3-MA_4377-R1	pASK-IBA3 derivate, coding region of truncated cytosolic MA_4377 (only R1 domain) from *M. acetivorans* at *Xba*I/*Nco*I with C-terminal Strep-tag II, *tet* promoter, Amp^R	This study
pASK-IBA3-MA_4377-R2	pASK-IBA3 derivate, coding region of truncated cytosolic MA_4377 (only R2 domain) from *M. acetivorans* at *Xba*I/*Nco*I with C-terminal Strep-tag II, *tet* promoter, Amp^R	This study
pASK-IBA3-MA_0863(rdmS)$_{O216K}$	pASK-IBA3 derivate, coding region of MA_0863 (rdmS) with amino acid exchange (Pyl216 to Lys) from *M. acetivorans* at *Sac*II/*Pst*I with C-terminal Strep-tag II, *tet* promoter, Amp^R	(Kwiatkowski, 2013)
pASK-IBA3-MA_2082	pASK-IBA3 derivate, coding region of MA_2082 from *M. acetivorans* at *Bam*HI/*Nco*I with C-terminal Strep-tag II, *tet* promoter, Amp^R	This study
pASK-IBA3-MA_2013	pASK-IBA3 derivate, coding region of MA_2013 from *M. acetivorans* at *Bam*HI/*Nco*I with C-terminal Strep-tag II, *tet* promoter, Amp^R	This study
pACYC-Duet1-MA_2013-PK	pACYCDuet-1 derivate, coding region of truncated MA_2013 (without R1 and HPt) from *M. acetivorans* at *Bam*HI/*Nde*I with N-terminal His-tag, T7 promoter, Cm^R	This study
pASK-IBA3-MA_2013-R1	pASK-IBA3 derivate, coding region of truncated MA_2013 (only R1) from *M. acetivorans* with C-terminal Strep-tag II, *tet* promoter, Amp^R	This study
pASK-IBA3-MA_2013-HPT	pASK-IBA3 derivate, coding region of truncated MA_2013 (only HPt) from *M. acetivorans* with C-terminal Strep-tag II, *tet* promoter, Amp^R	This study
pACYC-Duet1-MA_0016	pACYCDuet-1 derivate, coding region of MA_0016 from *M. acetivorans* at *Bam*HI/*Nde*I with N-terminal His-tag, T7 promoter, Cm^R	This study
pACYC-Duet1-MA_0018	pACYCDuet-1 derivate, coding region of MA_0018 from *M. acetivorans* at *Bam*HI/*Nde*I with N-terminal His-tag, T7 promoter, Cm^R	This study
pACYC-Duet1-MA_1268	pACYCDuet-1 derivate, coding region of MA_1268 from *M. acetivorans* at *Bam*HI/*Nde*I with N-terminal His-tag, T7 promoter, Cm^R	This study

pACYC-Duet1-MA_1269	pACYCDuet-1 derivate, coding region of MA_1269 from *M. acetivorans* at *Bam*HI/*Nde*I with N-terminal His-tag, T7 promoter, Cm^R	This study
pACYC-Duet1-MA_1366	pACYCDuet-1 derivate, coding region of MA_1366 from *M. acetivorans* at *Bam*HI/*Nde*I with N-terminal His-tag, T7 promoter, Cm^R	This study
pACYC-Duet1-MA_1468	pACYCDuet-1 derivate, coding region of MA_1468 from *M. acetivorans* at *Bam*HI/*Nde*I with N-terminal His-tag, T7 promoter, Cm^R	This study
pACYC-Duet1-MA_1469	pACYCDuet-1 derivate, coding region of MA_1469 from *M. acetivorans* at *Bam*HI/*Nde*I with N-terminal His-tag, T7 promoter, Cm^R	This study
pACYC-Duet1-MA_2445	pACYCDuet-1 derivate, coding region of MA_2445 from *M. acetivorans* at *Bam*HI/*Nde*I with N-terminal His-tag, T7 promoter, Cm^R	This study
pACYC-Duet1-MA_2012	pACYCDuet-1 derivate, coding region of MA_2012 from *M. acetivorans* at *Bam*HI/*Nde*I with N-terminal His-tag, T7 promoter, Cm^R	This study
pACYC-Duet1-MA_2861	pACYCDuet-1 derivate, coding region of MA_2861 from *M. acetivorans* at *Bam*HI/*Nde*I with N-terminal His-tag, T7 promoter, Cm^R	This study
pACYC-Duet1-MA_3068	pACYCDuet-1 derivate, coding region of MA_3068 from *M. acetivorans* at *Bam*HI/*Nde*I with N-terminal His-tag, T7 promoter, Cm^R	This study
pACYC-Duet1-MA_4376 (R3)	pACYCDuet-1 derivate, coding region of MA_4376 from *M. acetivorans* at *Sac*I/*Sal*I with N-terminal His-tag, T7 promoter, Cm^R	(Sexauer, 2021)
pACYC-Duet1-MA_4671	pACYCDuet-1 derivate, coding region of MA_4671 from *M. acetivorans* at *Bam*HI/*Nde*I with N-terminal His-tag, T7 promoter, Cm^R	This study
pET21a(+)-MA_4375 (*msrX*)	pET21a (+) derivate, coding region of MA_4375 from *M. acetivorans* with C-terminal His-tag, T7 promoter, Amp^R	(Sexauer, 2021)

Table S3: Oligonucleotids used in this study

Oligonucleotids	Sequence (5' → 3')
**Oligonucleotids for sequencing**	
pASK-IBA3-seq-fwd	CGCAGTAGCGGTAAACG
pASK-IBA3-seq-rev	GAGTTATTTTACCACTCCCT
pACYC-duet1-seq-fwd	TCTCCCTTATGCGACTCCTG
pACYC-duet1-seq-rev	GGGTTATGCTAGTTATTGCTCAGC
**Oligonucleotids for expression plasmids**	
pASK-IBA3-MA_4377-fwd	GCTATCCGCGGCATGAATGTGAGTAGAAAAATTCT
pASK-IBA3-MA_4377-rev	GCATACCATGGCCTCTTCGACAATAAGAATTTCTTTC
pASK-IBA3-MA_4377-CHPK-fwd	ATTCGAGCTCGGTACCCGGGATATGAATGTGAGTAGAAAAATTC
pASK-IBA3-MA_4377-CHPK-rev	GTGGCTCCAAGCGCTGAGACTCTGAGCTTCCCTATTTTC
pASK-IBA3-MA_4377-PKR1R2-fwd	GCTATCCGCGGCAGGTTGAATTCCGATAAGGTTAA
pASK-IBA3-MA_4377-PKR1R2-rev	GCATACCATGGCCTCTTCGACAATAAGAATTTCTTTC
pASK-IBA3-MA_4377-PKR1-fwd	ATTCGAGCTCGGTACCCGGGCTAGGTTGAATTCCGATAAG
pASK-IBA3-MA_4377-PKR1-rev	GTGGCTCCAAGCGCTGAGACGGAAACTGAACTTACCTG
pASK-IBA3-MA_4377-PK-fwd	GCTATCCGCGGCAGGTTGAATTCCGATAAGGTTAA
pASK-IBA3-MA_4377-PK-rev	GCGCCATGGCCTGTGAGGGGAATTG
pASK-IBA3-MA_4377-PK für QC	ATTCGAGCTCGGTACCCGGGCTATGAGGTTGAATTCCG
pASK-IBA3-MA_4377-PK für QC	GTGGCTCCAAGCGCTGAGACGCTTTGTGAGGGGAATTG
pASK-IBA-MA_4377-R1-fwd	ATGAATAGTTCGACAAAAATTAGTCTTGTGCTTGTAGTC
pASK-IBA3-MA_4377-R1-rev	GTGGCTCCAAGCGCTGAGACTGAAACTGAACTTACCTG
pASK-IBA3-MA_4377-R2-fwd	ATGAATAGTTCGACAAAAATTGGTAAGTTCAGTTTCGAC
pASK-IBA3-MA_4377-R2-rev	GTGGCTCCAAGCGCTGAGACTTCTTCGACAATAAGAATTTCTTTC
pASK-IBA3-MA_0863(rdmS)$_{O216K}$-fwd	GGACCGCGGAAAAAAGTTCGATATAAATC

pASK-IBA3-MA_0863(rdmS)$_{O216K}$-rev	GGACTGCAGTTATTTTTCGAACTCCGGGTGGCTCCAAGACTCTATTTGATTCATG
pASK-IBA3-MA_2082-fwd	ATTCGAGCTCGGTACCCGGGATATGGAAGGAAAACTGAGAAATTCTGGTATTGAG
pASK-IBA3-MA_2082-rev	GTGGCTCCAAGCGCTGAGACTCTCGACGCCCACAGGGA
pASK-IBA3-MA_2013-fwd	ATTCGAGCTCGGTACCCGGGAAGTGTGCGGTTTAAAAAAAC
pASK-IBA3-MA_2013-rev	GTGGCTCCAAGCGCTGAGACGACAGATCTGTTTTTCCAG
pACYC-duet1-MA_2013-PK-fwd	ACCATCATCACCACAGCCAGATGTGCGGTTTAAAAAAAC
pACYC-duet1-MA_2013-PK-rev	TATCCAATTGAGATCTGCCACTAGCATACTAACAGGCTTTC
pASK-IBA3-MA_2013-R1-fwd	ATTCGAGCTCGGTACCCGGGCGATGGTGAAAAGAGGGG
pASK-IBA3-MA_2013-R1-rev	GTGGCTCCAAGCGCTGAGACCTATACTTTCCAGGTCCAG
pACYC-duet1-MA_0016-fwd	ACCATCATCACCACAGCCAGATGGCAAGAGTAATGATC
pACYC-duet1-MA_0016-rev	TATCCAATTGAGATCTGCCATTAATTTGGTTCAGCAATTTTTTTAATTAC
pACYC-duet1-MA_0018-fwd	ACCATCATCACCACAGCCAGATGCCTGAAATCCTGATC
pACYC-duet1-MA_0018-rev	TATCCAATTGAGATCTGCCATCAACCAGGAGAATTCGTTG
pACYC-duet1-MA_1268-fwd	ACCATCATCACCACAGCCAGATGAAAACAAAGGTAGCTG
pACYC-duet1-MA_1268-rev	TATCCAATTGAGATCTGCCATTATTTTGACGGTAGTTTCAC
pACYC-duet1-MA_1269-fwd	ACCATCATCACCACAGCCAGATGGAAACATGGACGGCATTTAAAC
pACYC-duet1-MA_1269-rev	TATCCAATTGAGATCTGCCATTAAGTAAGATTTACGACTTCAAGCC
pACYC-duet1-MA_1366-fwd	ACCATCATCACCACAGCCAGATGCATAACATCAATCTTGAC
pACYC-duet1-MA_1366-rev	TATCCAATTGAGATCTGCCATCAAATCCGACTACGTCTC
pACYC-duet1-MA_1468-fwd	ACCATCATCACCACAGCCAGATGGACAAAGCAAAGATTC
pACYC-duet1-MA_1468-rev	TATCCAATTGAGATCTGCCATTATTCTTCAGTTACTTTACCC
pACYC-duet1-MA_1469-fwd	ACCATCATCACCACAGCCAGATGAAAAAAGCAAAAATTCTGGTTGTTG

pACYC-duet1-MA_1469-rev	TATCCAATTGAGATCTGCCATCATTCGTCGCTGTCGGAG
pACYC-duet1-MA_2012-fwd	ACCATCATCACCACAGCCAGATGAAAGTTCTGATTGCC
pACYC-duet1-MA_2012-rev	TATCCAATTGAGATCTGCCATTAAAAGCCCAGCTTTTTTC
pACYC-duet1-MA_2445-fwd	ACCATCATCACCACAGCCAGATGTTGAATGAGAAAAACAAAC
pACYC-duet1-MA_2445-rev	TATCCAATTGAGATCTGCCATTATGCTGCTTTTATGATAAATTC
pACYC-duet1-MA_2861-fwd	ACCATCATCACCACAGCCAGATGAAAGTACTGCTTGTAG
pACYC-duet1-MA_2861-rev	TATCCAATTGAGATCTGCCATTAACCTGAACCATTTTTAAC
pACYC-duet1-MA_3068-fwd	ACCATCATCACCACAGCCAGATGGCAAAAGTCTTGATTG
pACYC-duet1-MA_3068-rev	TATCCAATTGAGATCTGCCATTATGCACCGATTACTTTC
pACYC-duet1-MA_4376(R3)-fwd	GCGTCGAGCTCGAAAGAAATTCTTATTGTCG
pACYC-duet1-MA_4376(R3)-rev	CCCTTAGTCGACTTCTCCCAGATATTTATCG
pACYC-duet1-MA_4671-fwd	ACCATCATCACCACAGCCAGATGAAAAGTATACTTGTAGTTGAG
pACYC-duet1-MA_4671-rev	TATCCAATTGAGATCTGCCATCACAATACCTGGTAACC
pET21a(+)-MA_4375(msrX)-fwd	CTTTAAGAAGGAGATATACAATGAAAATATCACTTATTGACCTG
pET21a(+)-MA_4375(msrX)-rev	AGTGGTGGTGGTGGTGGTGCTTTATTTTGTCCAGCCGC
**Oligonucleotids for site directed mutagenesis**	
MA_4377-H497Q-fwd	GAGTTCCTTGCCAATATGAGCCAGGAGCTCCGGACGCCTCTG
MA_4377-H497Q-rev	CAGAGGCGTCCGGAGCTCCTGGCTCATATTGGCAAGGAACTC
MA_4377-D818N-fwd	CCTGATGTTATCACTCTGAATGTTTTACTTCCCGATACTAGTGGC
MA_4377-D818N-rev	GCCACTAGTATCGGGAAGTAAAACATTCAGAGTGATAACATCAGG
MA_4377-D941N-fwd	CAGCAGCCGGATATCCTCATCCTCAACCTGCTGATGCCCGAGATAAGT
MA_4377-D941N-rev	ACTTATCTCGGGCATCAGCAGGTTGAGGATGAGGATATCCGGCTGCTG

Table S4: Sequences of 53 putative HK and 15 RR of M. acetivorans. The protein name corresponds to the old locus tag. The new locus tag and the acession number of the protein in the uniprot database is displayed in the table. For the Alignment (Figure S2) and the phylogenetic tree (Figure 1A) the aminoacid sequence of the HisKA domain was employed. For the alignment (Figure S3) the amino acid sequence of the REC domain was employed.

Protein	New locus tag	Organism	Accession number	database
MA0014	MA_RS00065	*M. acetivorans*	Q8TUQ1	UniProt
MA0203	MA_RS01090	*M. acetivorans*	Q8TU70	UniProt
MA0490	MA_RS02555	*M. acetivorans*	Q8TTE7	UniProt
MA0551	MA_RS25000	*M. acetivorans*	Q8TT86	UniProt
MA0552	MA_RS02905	*M. acetivorans*	Q8TT85	UniProt
MA0619	MA_RS03260	*M. acetivorans*	Q8TT21	UniProt
MA0620	MA_RS03265	*M. acetivorans*	Q8TT20	UniProt
MA0758	MA_RS03960	*M. acetivorans*	Q8TSN7	UniProt
MA0759	MA_RS03965	*M. acetivorans*	Q8TSN6	UniProt
MA0777	MA_RS04070	*M. acetivorans*	Q8TSL9	UniProt
MA0863 (RdmS)	MA_RS04495	*M. acetivorans*	Q8TSD5	UniProt
MA0970	MA_RS24390	*M. acetivorans*	Q8TS36	UniProt
MA1149	MA_RS05985	*M. acetivorans*	Q8TRM6	UniProt
MA1267	MA_RS06580	*M. acetivorans*	Q8TRB3	UniProt
MA1270	MA_RS06595	*M. acetivorans*	Q8TRB0	UniProt
MA1274	MA_RS06615	*M. acetivorans*	Q8TRA6	UniProt
MA1322	MA_RS06850	*M. acetivorans*	Q8TR62	UniProt
MA1463	MA_RS24430	*M. acetivorans*	Q8TQS7	UniProt
MA1470	MA_RS07625	*M. acetivorans*	Q8TQS0	UniProt
MA1627	MA_RS08450	*M. acetivorans*	Q8TQC1	UniProt
MA1628	MA_RS08455	*M. acetivorans*	Q8TQC0	UniProt
MA1630	MA_RS08465	*M. acetivorans*	Q8TQB8	UniProt
MA1645	MA_RS08530	*M. acetivorans*	Q8TQA5	UniProt
MA1646	MA_RS08535	*M. acetivorans*	Q8TQA4	UniProt
MA1704	MA_RS08850	*M. acetivorans*	Q8TQ48	UniProt
MA1739	MA_RS09030	*M. acetivorans*	Q8TQ13	UniProt
MA1844	MA_RS24495	*M. acetivorans*	Q8TPR2	UniProt
MA1878	MA_RS09795	*M. acetivorans*	Q8TPM8	UniProt
MA1957	MA_RS10200	*M. acetivorans*	Q8TPF6	UniProt
MA1991	MA_RS24525	*M. acetivorans*	Q8TPC2	UniProt
MA2013	MA_RS10480	*M. acetivorans*	Q8TPA1	UniProt
MA2082	MA_RS10815	*M. acetivorans*	Q8TP40	UniProt
MA2256	MA_RS11710	*M. acetivorans*	Q8TNM8	UniProt
MA2266	MA_RS11765	*M. acetivorans*	Q8TNL9	UniProt
MA2294	MA_RS11915	*M. acetivorans*	Q8TNJ1	UniProt
MA2348	MA_RS12170	*M. acetivorans*	Q8TNE0	UniProt
MA2553	MA_RS13275	*M. acetivorans*	Q8TMU8	UniProt
MA2555	MA_RS13285	*M. acetivorans*	Q8TMU6	UniProt
MA2732	MA_RS14295	*M. acetivorans*	Q8TMC7	UniProt
MA2757	MA_RS28770	*M. acetivorans*	Q8TMA7	UniProt
MA2784	MA_RS28790	*M. acetivorans*	Q8TM82	UniProt
MA2890	MA_RS15160	*M. acetivorans*	Q8TLY2	UniProt

MA3066	MA_RS16035	*M. acetivorans*	Q8TLH0	UniProt
MA3346	MA_RS17465	*M. acetivorans*	Q8TKQ3	UniProt
MA3368	MA_RS25835	*M. acetivorans*	Q8TKN3	UniProt
MA3370	MA_RS24765	*M. acetivorans*	Q8TKN1	UniProt
MA3405	MA_RS17780	*M. acetivorans*	Q8TKK0	UniProt
MA3481	MA_RS18195	*M. acetivorans*	Q8TKC7	UniProt
MA3543	MA_RS24775	*M. acetivorans*	Q8TK73	UniProt
MA3962	MA_RS20675	*M. acetivorans*	Q8TJ26	UniProt
MA4026	MA_RS21010	*M. acetivorans*	Q8TIW4	UniProt
MA4377	MA_RS22890	*M. acetivorans*	Q8THY1	UniProt
MA4561 (MsmS)	MA_RS23780	*M. acetivorans*	Q8THF6	UniProt
MA_0016	MA_RS00075	*M. acetivorans*	Q8TUP9	UniProt
MA_0018	MA_RS00090	*M. acetivorans*	Q8TUP7	UniProt
MA_1268	MA_RS06585	*M. acetivorans*	Q8TRB2	UniProt
MA_1269	MA_RS06590	*M. acetivorans*	Q8TRB1	UniProt
MA_1366	MA_RS07090	*M. acetivorans*	Q8TR18	UniProt
MA_1468	MA_RS07615	*M. acetivorans*	Q8TQS2	UniProt
MA_1469	MA_RS07620	*M. acetivorans*	Q8TQS1	UniProt
MA_2012	MA_RS10475	*M. acetivorans*	Q8TPA2	UniProt
MA_2861	MA_RS15020	*M. acetivorans*	Q8TM09	UniProt
MA_3068	MA_RS16045	*M. acetivorans*	Q8TLG8	UniProt
MA_4376	MA_RS22885	*M. acetivorans*	Q8THY2	UniProt
MA_4671	MA_RS11705	*M. acetivorans*	Q8TNM9	UniProt
MA_2445	MA_RS12700	*M. acetivorans*	Q8TN48	UniProt
MA_0015	MA_RS00070	*M. acetivorans*	Q8TUQ0	UniProt
MA_3057	MA_RS15990	*M. acetivorans*	Q8TLH9	UniProt

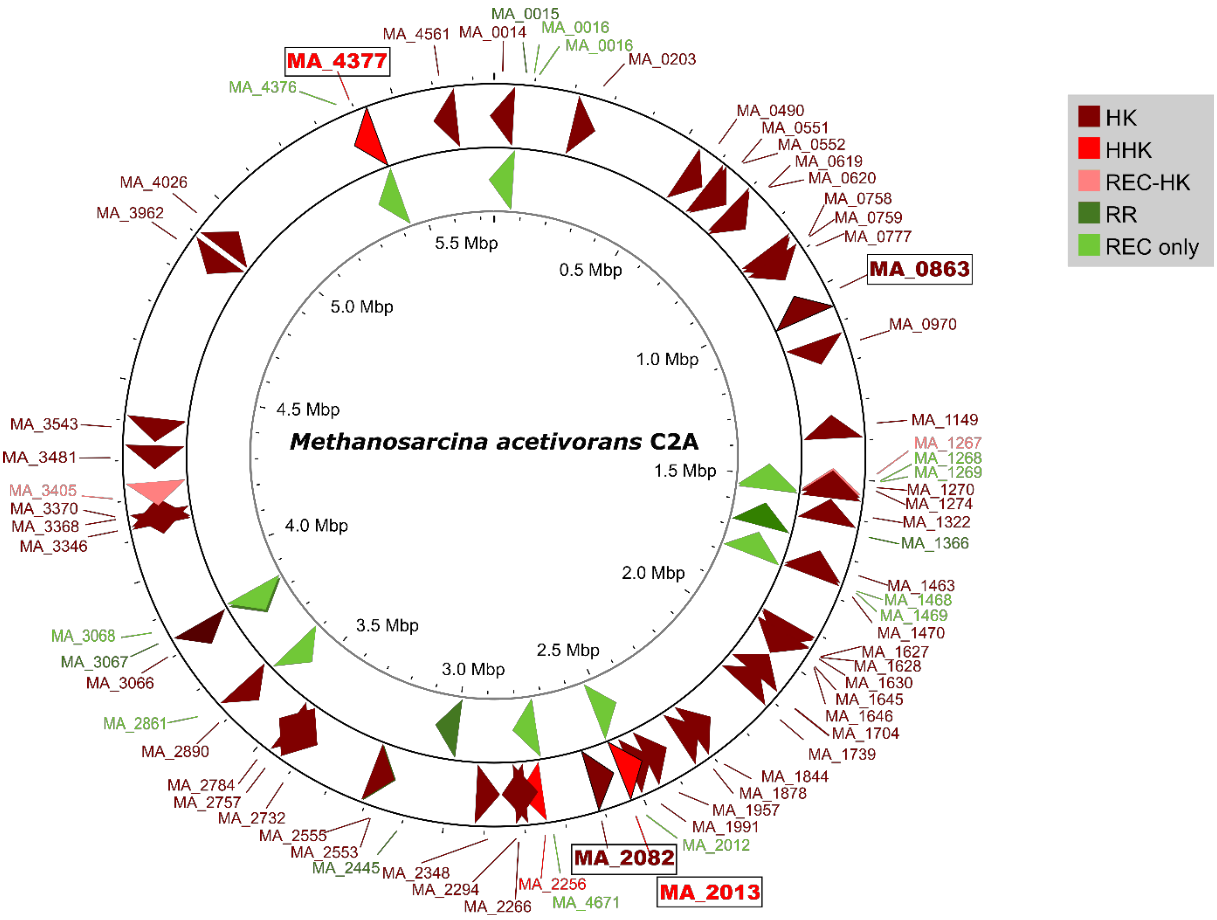

Figure S1: Genomic distribution of HK and RR in the genome of M. acetivorans. Genomic distribution of histidine kinases (HK = Histidine Kinase, HHK = hybrid histidine kinase, REC-HK = histidine kinase with N-terminal REC domain) and response regulators (RR = response regulator with output domain, REC only = response regulator with only REC domain) in the genome of *M. acetivorans*. The genome of M. acetivorans (NC_003552.1) was obtained from the NCBI database and analysed using the online tool PROKSEE (https://proksee.ca/) (Grant *et al.*, 2023).

```
MA_type 1A  MA3405  NM SH-------ELKTP LNSIIG----F----SDLLKEEIAGPLNEKQSRYVQFISSSGKN 45
            MA2294  SM SH-------ELRTP LNSIIG----F----SDMLLTQNFGPLNKKQLRYVNNISVSGNH 45
            MA3962  NM SH-------ELRTP LNSVIG----F----SDLLLEGAFGPLNTKQSKYVNNILISGKN 45
            MA1149  NM SH-------ELRTP LNAVIG----F----SDLLLSETAGPLNEKQKRYTENISKSGSH 45
            MA2555  NM SH-------ELRTP LNSIIG----F----SDLLYEKIYGDLNEKQLKAVGNISRSGKH 45
            MA1739  NI SH-------ELRTP LNSIIG----F----SDLLCEQIFGELNEKQLRYAGNISKSGKH 45
            MA3368  NM SH-------ELRTP LNSIIG----F----SDMLYEQAYGELNRKQLRAIGNISSSGKH 45
            MA2553  NM SH-------ELRTP LNSIIG----F----SDLLYEKVYGELNLKQTKAVGNISNSGKH 45
            MA4377  NM SH-------ELRTP LNSIIG----F----SDILIERVFGELNEKQLKYVNNISGSGKH 45
            MA2348  NM SH-------ELRTP LNAVIG----F----ADILNEEGFGPLNKKQKRFVGNISTSGKH 45

no type     MA0777  -- -------------- ------------------------------------------- 0

MA_type 1B  MA1957  TV SH-------ELKTP LNSIIG----F----SDLLLEDSSGKLSERQARYINNISISGKH 45
            MA2256  TM SH-------ELRTP LTAIIG----F----SELMLGEATGEFDELNRKFLGHISNSGKH 45
            MA2013  NM SH-------EIRTP MNAVLG----M----LEMLLETS---LTDEQREYLQLAHASAES 42

MA_type 1C  MA1270  VS SH-------DLQEP LRMIAS----Y----LQLLQRRYQGELDERADKYIYFAVDGASR 45

MA_type 2   RdmS    -- -------------- --------------------------EDQQQKAIDTVINSSER 17
            MsmS    EF VE-------EMMFP EK--AE----Y----GEIMDYETLYAIDSQQQKAVNTFIHYSEK 43

MA_type 3   MA0203  EI HH-------RIKNN LQ--------VISSLLDLQAEKFNSREDIKDSEVLEAFRESQDR 45
            MA0490  EI HH-------RIKNN LQ--------VISSLLSLEAEKFSDE------KMLESFRESQNR 39
            MA0551  EI HH-------RIKNN LQ--------VISSLLDLQAEKFQN------KEVLEAFRESQNR 39
            MA0552  EI HH-------RIKNN LQ--------VISSLLDLQAEKFNNREDIKDSEILEAFRESQDR 45
            MA0619  EI HH-------RIKNN LQ--------VISSLLSLEAEKFSDE------RTLEAFRESQNR 39
            MA0620  EI HH-------RIKNN LQ--------VISSLLSLQAEKFEDR------EVIEAFRESQNR 39
            MA0758  EI HH-------RIKNN LQ--------VISSLLDLQAEKFRD------KDVLEAFRESQSR 39
            MA0759  EI HH-------RIKNN LQ--------VISSLLDLQAEKFRD------KEVLEAFRESQSR 39
            MA0970  EI HH-------RIKNN LQ--------VISSLLDLQAEKFRGKKNIEDSKILEAFKESQDR 45
            MA1274  EI HH-------RIKNN LQ--------VISSLLDLQAEKFRSREHVEDSEVLNAFKESQER 45
            MA1322  EI HH-------RIKNN LQ--------VISSLLDLQAEKFGNKKYIMNSEVMDAFRESQDR 45
            MA1470  EI HH-------RIKNN LQ--------VISSLLELQAEKFDNL------EVLEAFRESQNR 39
            MA1627  EI HH-------RIKNN LQ--------VISSLLDLQADKFDNP------KVIEAFRESQNR 39
            MA1628  EI HH-------RIKNN LQ--------IISSLLDLQAEMFKGRSNIRDSEVLKAFAVSMDR 45
            MA1630  EI HH-------RIKNN LQ--------IISSLLDLQAEKFED------KNVTEAFREGQNR 39
            MA1645  EI HH-------RIKNN LQ--------VISSLLDLQAEQFKNRENIKDSEVLEAFRESQDR 45
            MA1646  EL HH-------RIKNN LQ--------VISSLLDLQADLFKGKKTITDSEVLKAFNESIDR 45
            MA1704  EI NH-------RIKNN LQ--------VISSLLSFEAEKSTDP------EILEAFRETQNR 39
            MA1844  EI HH-------RIKNN LQ--------VISSLLELQADKFKDR------EVIEAFRESQNR 39
            MA1878  EI HH-------RIKNN LQ--------VISSLLDLQIDIFSNREICKTPEVIEAFRESQNR 45
            MA1991  EI HH-------RIKNN LQ--------VISSLLSLQAEKFRD------QEVLEAFRESQDR 39
            MA2082  EI HH-------RIKNN LQ--------VISSLLDLQAEKFRD------KEVLEAFRESQNR 39
            MA2266  EI HH-------RIKNN LQ--------VISSLLSLQAEYFSDP------KVKESFKDSQNR 39
            MA2732  EI HH-------RIKNN LQ--------VISSLLDLECDSLLSG-TPDHKKIAEAFRESHNR 44
            MA2757  EI HH-------RIKNN LQ--------VISSLLDLQAEQFKNRECIKNSEVLEAFRESQAR 45
            MA2784  EI HH-------RIKNN LQ--------VISSLLDLQAEKFKDREDIKDSEVLEAFRESQDR 45
            MA3346  EI HH-------RIKNN LQ--------VISSLLDLQAENFSKHEVCKTPKVVEAFKESQDR 45
            MA3370  EI HH-------RIKNN LQ--------VISSLLDLQAGKFNNKEHIRDSEVLEAFKESQDR 45
            MA3481  EI HH-------RIKNN LQ--------VISSMLSLQAEKFSDE------ETLEAFRESQNR 39
            MA3543  EI HH-------RIKNN LQ--------VISSLLDLQAEKFNKREGIKDSEVMEAFRESQDR 45
            MA4026  EI HH-------RIKNN LQ--------VISSLLDLQAEKFSHREAVPTLEILEAFKESQNR 45
            MA1267  EI HH-------RIKNN LQ--------VISSLLDLQAEKFED------PTIRQAFRESQNR 39
            MA1463  EI NH-------RIKNN LQ--------IVSSLLDLQAEQFSDK------KVIEAFRESENR 39
            MA2890  EI HH-------RIKNN LQ--------IVSSLLSLQADKFKDK------DVIEAFRESENR 39

MA_CheA     MA0014  -- -------------- ---------RISTEQLDKL-MNLVGELVINRSRVKELTGESKSK 34
            MA3066  GS SHHFSESAASAKTP LETQRQETIRVRTSNLDNI-MNLVGELVINKGRLLQISQEYNLP 59
```

Figure S2: Partial amino acid sequence alignment of HisKA domain of all putative HK of *M. acetivorans*. Acession numbers of the employed sequences are listed in Supplement Table S4, for the alignment only the amino acid sequences of the HisKA domain were taken. The Alignment was constructed using CUSTAL Omega and modified using PowerPoint. Conserved His residue is highlighted in bold with gray backgound. H-box region groups into four distinct groups (Ma_type 1 A/B/C, Ma_type 2, Ma_type 3 and MA_CheA). MA_type 1 is similar to the Type I HK of bacteria. As the amino acid arrangement differs within the group, the subgroups A, B and C were created. MA_type 2 kinases

are characterized by the absence of an H-box and MA_type 3 kinases cannot be grouped with a known type of bacterial kinases. MA_CheA are highly similar to bacterial CheA proteins.

```
MA0016         --------MARVMIVDDAEFMRMVIRDILLKHGHE-VVAEVGDGEEAIQTYL-------E  44
MA0018         --------MPEILIVEDNLLNLVIEADLLKSCGY--DPKKAKNGFEALEVLS------KV  44
MA1268         MKTKVAAKPIEILLVEDSEGDVGLIEEVFEEAKIRNNLHIVEDGEEAILFLRGEKQFSGI  60
MA1269         METWTAFKPVDILLVEDNKGDVGLIEEVFESSKVRNKLYVVEDGEEAVHFLLREGKFSDV  60
MA1366         ----------KILIMGNGNNVHNNLQKVLEAENY--NVVSASDNFSAIETV-------NE  41
MA1468         ------MDKAKILVVEDQNIVALNLRNRLKNMGYI-VPTIAISGEEAIRKTE-------L  46
MA1469         ------MKKAKILVVEDQNIVALNIRNKLKNLGYT-VPGTASTGEEAIRKAE-------L  46
MA2012         ----------KVLIAEDEPISNLWLKNTLTRWGY--EAISTRDGYEAWEVLN------ES  42
MA2013-R1      ----------NVLFAEDHPINQKLILGLLEKKGH--KLTLVTSGKDALDALS------RR  42
MA2445         ----------KVLIVDDKKENVELMEAYLAVEPY--DVITAYGGKEAFQKV-------KE  41
MA2861         ---------MKVLLVDDDPVFLELSKTFLEVFHDI-NSDTVESARQALEKLD-------E  43
MA3068         --------MAKVLIVDDTAFMRKLLKNILFGAGFD-IAGEAENGKQAVEMYK-------G  44
MA4376         --------MKEILIVEDNPMNMELILDLLEFYGH--RVTEAEDGIKALERLA------EK  44
MA4377-R1      ----------LVLVVDDDINSNELISVVLREAGY--STASLHNGKDVLEVA-------KK  41
MA4377-R2      ----------KVLIIDDDENAVELLSSMIESEGF--EIVKAYSGQAGLDKLF------SE  42
MA4671         ------MEMKSILVVEDSPVILELISFFLTSSGY--ESRETGDGFDALKIAE------EN  46
                          ::.  :           :

MA0016         VKPDLVLMDIIMP-DMDGKEALQKLLLID-----PDAKVVMCSSLGQQALITESMKIGAM  98
MA0018         KVD-LVLMDMELP-KMHGLELLQRIKCNP---ETQGIRVVAVTGHCDPESEQEFLKAGCH  99
MA1268         SRPDIILLDLNLP-KKDGREVLEEIKEDD---DLKNIPVVVLTTSKAEEDVLKSYNLHAN  116
MA1269         PRPGIILLDLNLP-KKDGREVLEEIKEDD---DLKNIPVVVLTTSRAEEDILESYKLHAN  116
MA1366         EKPDLVLLDTVYL-ETDGFEICRQLKDSP---RYWWIPIMMLSERNKTEDGIKAFDSGAD  97
MA1468         TTPDLVLMDIMLKGDMDGIEAARIIKSRF------SAPVIYLTACTDIGILERAKLTEPA  100
MA1469         TNADLVLMDIMLKGDMDGIEAAREIKARL------KIPVLYLTAYTDDETLERAKMTEPA  100
MA2012         DFPRVVILDWEMP-KMKGIEVCEKIKKDP---RLSSIYIILITGRDLTEDMEAGFKAGAD  98
MA2013-R1      DFD-AVLMDIQMP-GMDGLEATRRIRDPSSGVRRHNIPIIAFTARALKEDREKCFEAGMN  100
MA2445         EKPDIILLDVMMP-EVNGYEVCKILKGNP---ETQFIPVLMLTALSELEDRIRGIEVGAD  97
MA2861         LSYDVVVSDYDMP-YMDGISFLKTIRDKR-----INIPFILFTGVGKEEIKSQAIENGVD  97
MA3068         LKPDVVTMDVVMP-EMTGIDALKQIKALD-----KDAKIVMCTAIGQENIVKTAIKLGAR  98
MA4376         KFD-IILLDMQLP-KMDGLEVLDRIKKNP---ATADIPVIAVTAHAMKGSEEHFIEMGCV  99
MA4377-R1      LKPDVITLDVLLP-DTSGWNVLKQLKSDL---DTTSIPVLIISVTDNNE---LGVALGAT  94
MA4377-R2      QQPDILILDLLMP-EISGFEIISRLRDGE---QTKDIPLIVCTAGEFTEKNIEKLNGELK  98
MA4671         RFD-LILLDKQLP-GFDGLEVLKKIKKIF---EIRKTSVIALMAHAMQGDEDRFLKAGCN  101
                    :  *        * .    :             .:

MA0016         GF-----IIKPFEPDGMLDVIKKIAEPN---------------  121
MA0018         AV-----LSKPINFDLFGAQVKEFLTATNSPG-----------  126
MA1268         AY-----VTKPVDFDQFIRVIKSIEDFWLEVVKLPSK------  148
MA1269         AY-----VTKPVDFDQFIKVIKSIENFWLEVVNLT--------  146
MA1366         DY-----ITMPFNPLELKARVGMIL------------------  117
MA1468         GY-----ISKPFKEKDLVSNIEVALQKNKL-GKVTEE------  131
MA1469         GY-----ISKPFKEEDLHSNIEMALHKHRT-EKKEIENSDSDE  137
MA2012         DY-----LKKPFDNRKLKTKLDTAR------------------  118
MA2013-R1      YY-----ISKPLKKEKLLNILEDIR------------------  120
MA2445         DF-----LTKPINRLELKTRVKSLL------------------  117
MA2861         SL-----IQKRGDPKAQYSELSKRIWQIVKNGSG---------  126
MA3068         GY-----IIKPFQAPKVIEEIKKVIGA----------------  120
MA4376         DY-----ISKPIDIHRFRSLIDKYLGE----------------  121
MA4377-R1      YS-----FTKPVRRVELLDSLREIT------------------  114
MA4377-R2      GHLISIMKKGTFGRKELINRIKQLA------------------  123
MA4671         GY-----ISKPIDIDRFKLILDTCTGGYQVL------------  127
                                   :
```

Figure S3: Amino acid sequence alignment of REC domain of all putative RR of *M. acetivorans*. Acession numbers of the employed sequences are listed in Supplement Table S4, for the alignment only the amino acid sequences of the REC domain were considered. The Alignment was constructed using CUSTAL Omega and modified using PowerPoint. All proteins share a conserved Asp residue, highlighted in bold with gray background.

A.

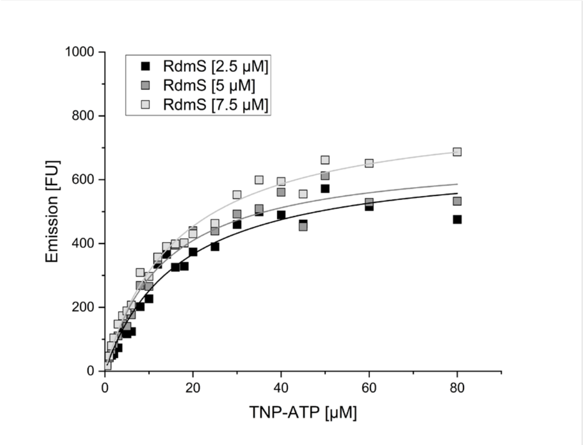

B.

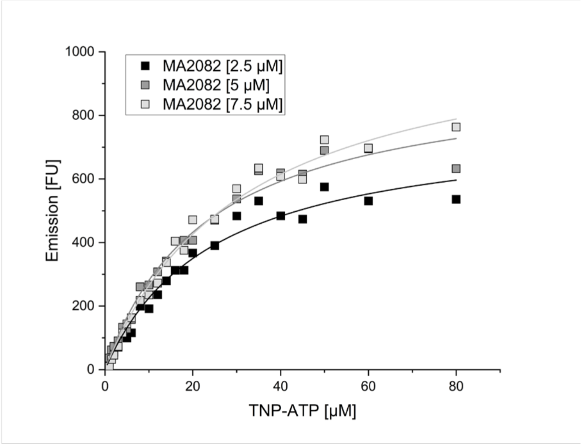

C.

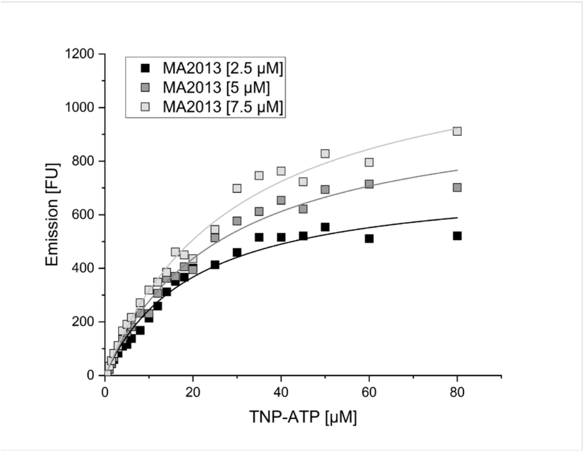

D.

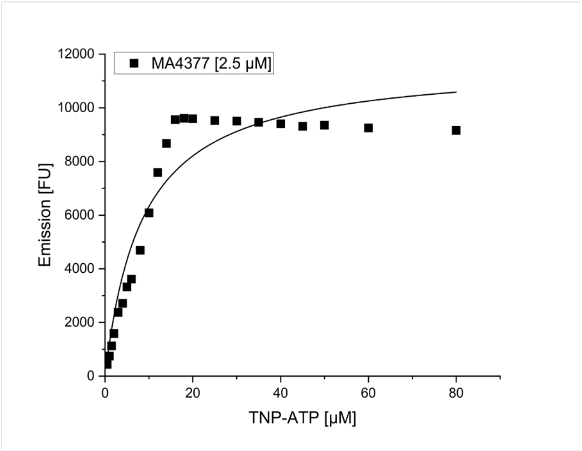

E.

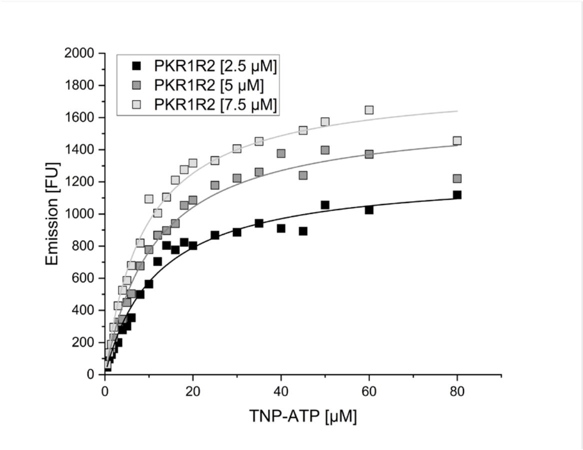

Figure S4: TNP-ATP association kinetics of putative HKs. Constant protein concentration (2.5 µM, 5 µM or 7.5 µM) was mixed with variable TNP-ATP concentration (0 – 80 µM) in kinase buffer. A fluorescence emission spectrum in the range of 450 - 650 nm was recorded using a fluorescence spectrometer (FP-8300 fluorescence spectrometer, Jasco) and a quartz cuvette (SUPRASIL® cuvette, 3x3 mm, Hellma Analytics). The excitation wavelength was 410 nm, and the slit width was 5 nm. The fluorescence of TNP-ATP was determined as a maximum at 541 nm. The Kd value was determined using the SOLVER function of Microsoft Excel. (**A.**) TNP-ATP association kinetics of RdmS. (**B.**) TNP-

ATP association kinetics of MA2082. (**C.**) TNP-ATP association kinetics of MA2013. (**D.**) TNP-ATP association kinetics of MA4377. (**E.**) TNP-ATP association kinetics of PKR1R2.

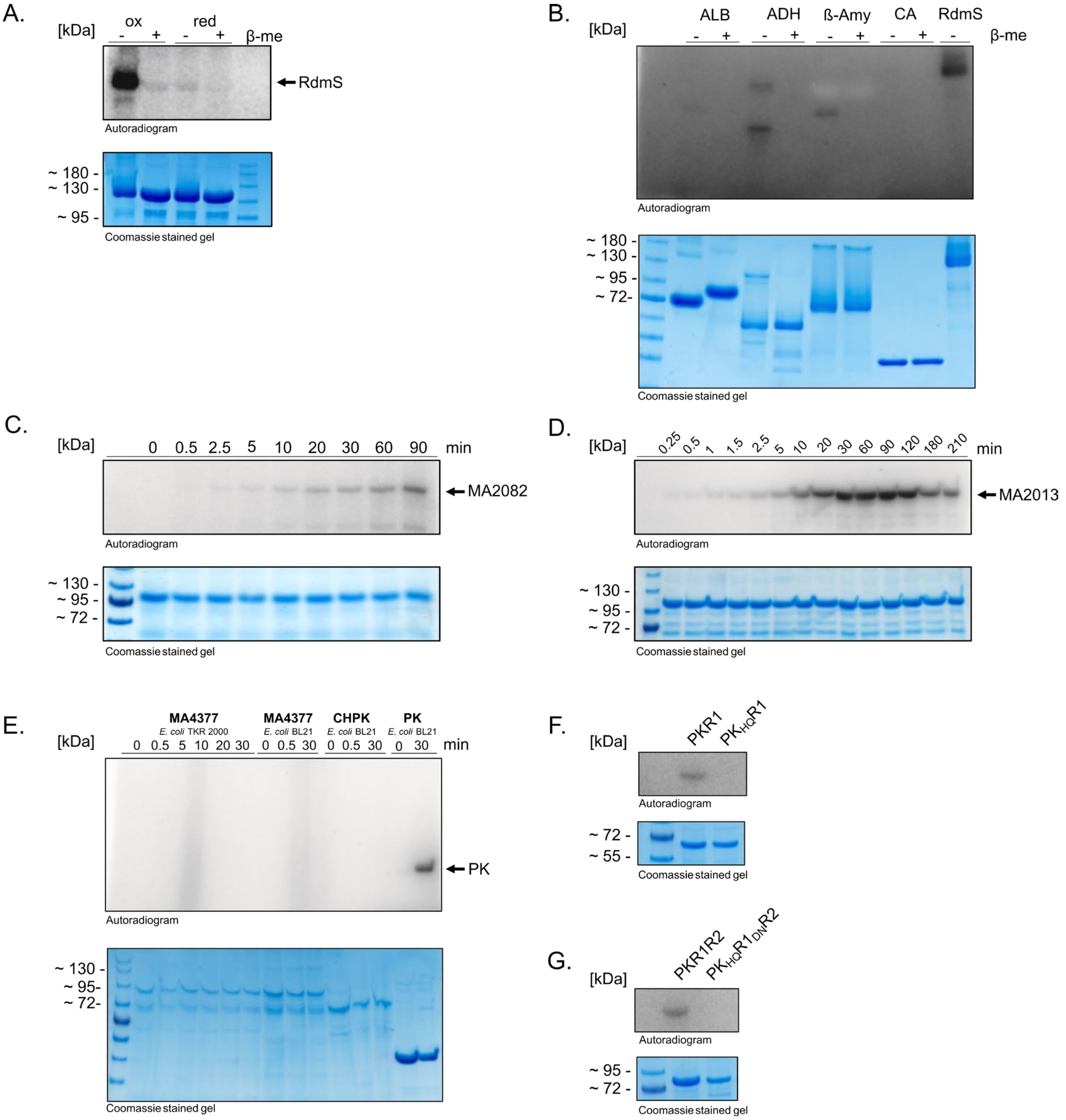

Figure S5: Autophosphorylation assay of different putative HK. (**A.**) Autophosphorylation assay of RdmS under oxidizing (ox) and reducing (red) conditions. 10 μM RdmS was incubated with [γ-^{32}P]-ATP and the reaction was stopped using 4x SDS sample buffer with (+) or without (-) β-mercaptoethanol (β-me). (**B.**) Autophosphorylation assay of standard proteins and RdmS under oxidizing conditions. 10 μM of the standard proteins albumin (ALB), alcohol dehydrogenase (ADH), β-amylase (β-Amy), carbonic anhydrase (CA) and RdmS were incubated with [γ-^{32}P]-ATP and the reaction was stopped using 4x SDS sample buffer with (+) or without (-) β-mercaptoethanol (β-me). (**C.**) Autophosphorylation Assay of MA2082. 10 μM MA2082 was incubated with [γ-^{32}P]-ATP and the reaction was stopped using 4x SDS sample buffer with β-mercaptoethanol. (**D.**) Autophosphorylation Assay of MA2013. 10 μM MA2013 was incubated with [γ-^{32}P]-ATP and the reaction was stopped using 4x SDS sample buffer with β-mercaptoethanol. (**E.**) Autophosphorylation Assay of MA4377 and truncated protein variants. MA4377 produced in different *E. coli* strains and truncated variants (CHPK and PK) were incubated with [γ-^{32}P]-ATP and the reaction was stopped using 4x SDS sample buffer with β-mercaptoethanol. (**F.**) Autophosphorylation assay of the MA4377 variants PKR1 and $PK_{H497Q}R1$. 10 μM protein was incubated with [γ-^{32}P]-ATP and the reaction was stopped using 4x SDS sample buffer with β-mercaptoethanol. (**G.**) Autophosphorylation assay of the MA4377 variants PKR1R2 and $PK_{H497Q}R1_{D818N}R2$. 10 μM protein was incubated with [γ-^{32}P]-ATP and the reaction was stopped using 4x SDS sample buffer with β-

mercaptoethanol. The samples of all assays were separated by SDS-PAGE and the radioactive signals detected by PhosphorImager (Autoradiogram). The same SDS gel was stained with Coomassie (Coomassie stained gel) to visualize the loaded protein.

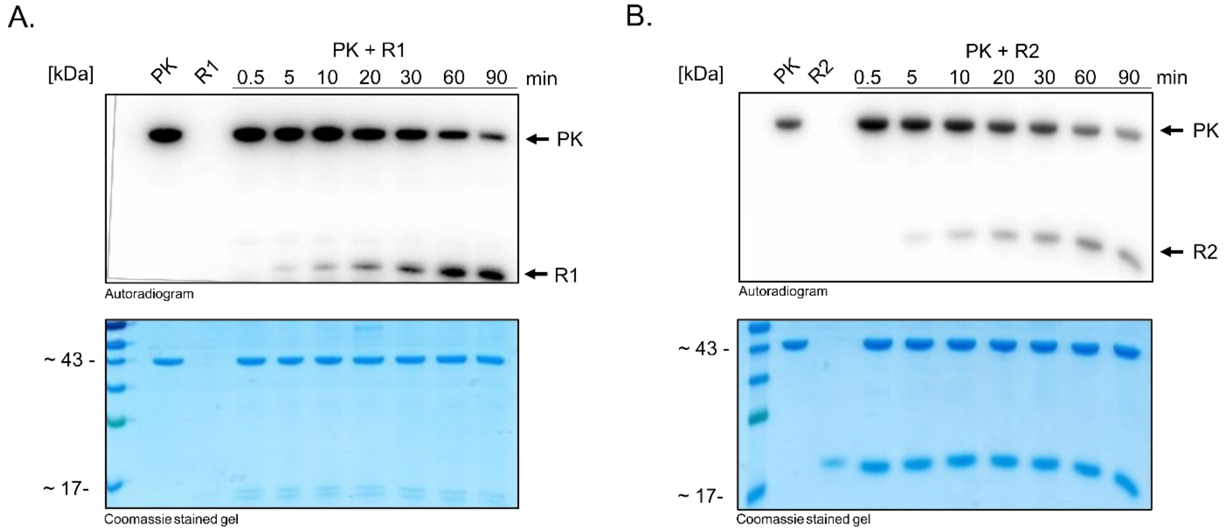

Figure S6: Transphosphorylation assays of PK. Autoradiogram and corresponding SDS-PAGE (Coomassie stained gel) of the transphosphorylation reaction of different protein variants. Purified recombinant PK (10 µM) was phosphorylated with [γ-^{32}P]-ATP, after removing excessive ATP with illustra™ MicroSpin™ G-25 Columns (GE healthcare), the second protein was added in equimolar amount. (**A.**) Transphosphorylation assay with PK and R1. (**B.**) Transphosphorylation assay of PK with R2.

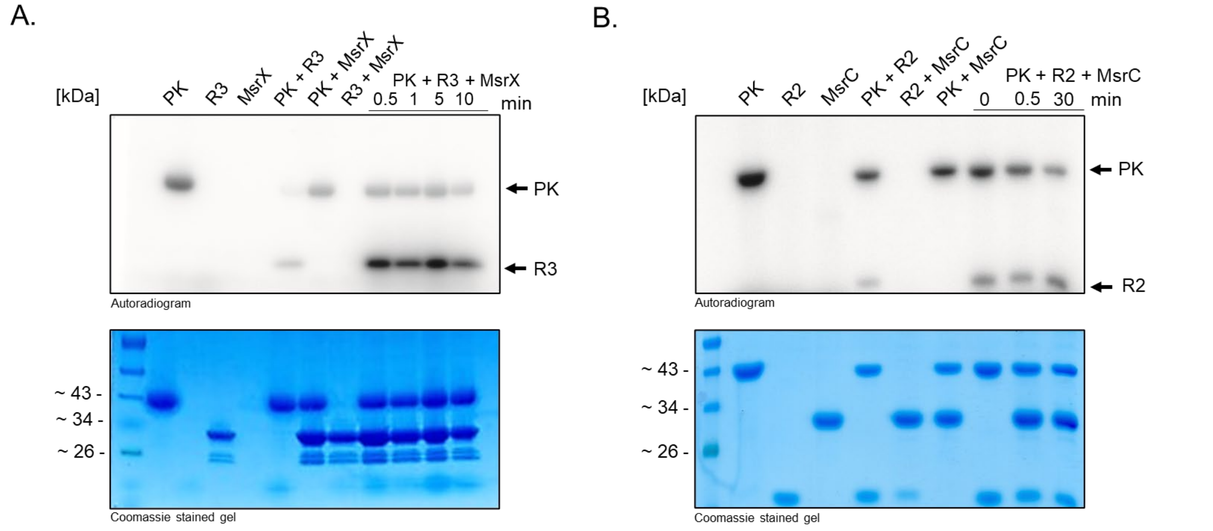

Figure S7: Putative further components of the phosphorelay of MA4377. (**A.**) Phosphorylation assay to test whether MsrX is receiving a phosphate from PK or R2. **T**ranscriptional regulator MsrX is not involved in a phosphorelay. (**B.**) Phosphorylation assay to test whether MsrC is receiving phosphate from PK or R3. Transcriptional regulator MsrC is not involved in a phosphorelay.

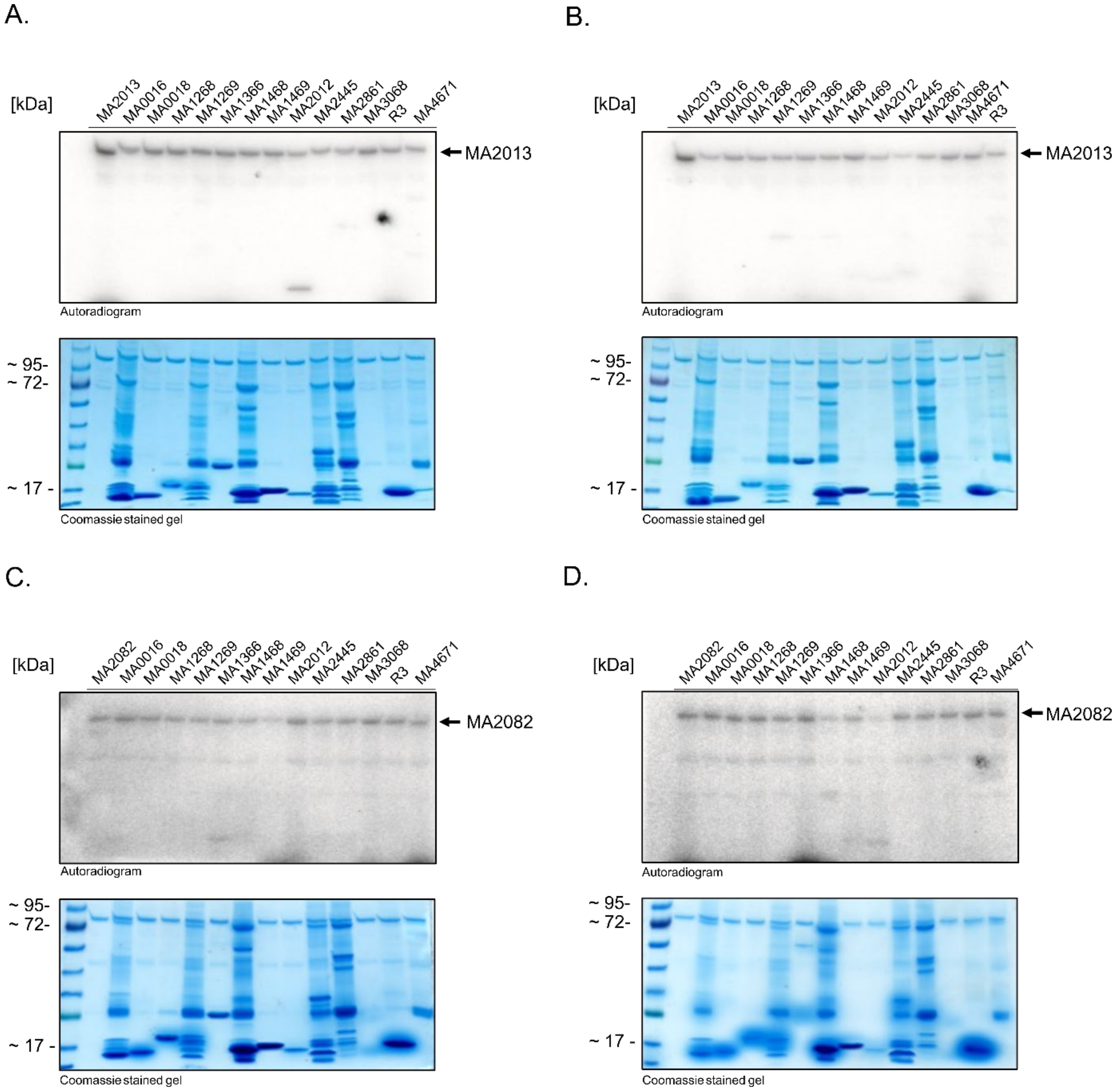

Figure S8: Phosphotransfer profiling of MA2013 and MA2082. The purified HKs MA2013 and MA2082 are incubated with radioactive labelled ATP and then incubated with each of the purified RRs. The reactions were analyzed by SDS-PAGE and PhosphorImager. (**A.**) Phosphotransfer profile of MA2013 after 30 seconds. Transfer is visible for the REC-only RR MA2012. (**B.**) Phosphotransfer profile after 60 minutes. Transfer of phosphate was not clearly identified, possible transfer to MA2445 and MA1268. A signal around 80 kDa corresponds to the autophosphorylated kinase, a second signal corresponds to the transphosphorylated RR. (**C.**) Phosphotransfer profile of MA2082 after 30 seconds. (**D.**) Phosphotransfer profile after 60 minutes. A signal around 80 kDa corresponds to the autophosphorylated kinase, a second signal corresponds to the transphosphorylated RR. Transfer is not clearly identified, MA1468, MA1469, MA2012 and MA1366 might be involved in the phosphorelay.

Amino acid sequences of HK domains of putative HK and amino acid sequences of REC domains of RR of *M. acetivorans*.

>MA3405
NMSHELKTPLNSIIGFSDLLKEEIAGPLNEKQSRYVQFISSSGKNLLEIINDILDLSKAESGEEDLNVEKFSVDESINKVISV
VLPQAQEKNIILNYQSENRTLWITADEGKFRQIMENLLSNAIKFTPAGGSIDVTLKQEGLLVTIEVKDTGIGIPEDSFEKIFK
PFIQIDSSLSRNFEGTGLGLTLVKKYVEMHGGNIYVESKIGEGSSFRFELPVTR
>MA2294
SMSHELRTPLNSIIGFSDMLLTQNFGPLNKKQLRYVNNISVSGNHLLKLINGILDLSKVEAGKMELKVEEFSVLDSISEVKVL
LTPLASKKDIQILSVVDKELTTIRADRTKFKQILYNLVDNAIKFTHEDGFVIVDARVEGDQAKIMVKDTGIGISKAGVKKIFQ
PFTQLENSEYGKQKGTGLGLSLVKKFVEMHTGKIWVESEFGEGSKFIFTIPLSL
>MA3962
NMSHELRTPLNSVIGFSDLLLEGAFGPLNTKQSKYVNNILISGKNLLEIINNLLDISRLEAGEKTLKYENVDIASLIGDVRMS
LLSPASVKRITVELKIDPSVGNVRADITKLRQILYNLVSNAIKFTPAKGKVVISACKKEGVLEVKVSDTGIGLSKDSHEKIFM
PFTQADSSAARGYGGAGLGLYIVRNFVDLHGGKIWVDSEVGKGSIFTFTLPAE
>MA1149
NMSHELRTPLNAVIGFSDLLLSETAGPLNEKQKRYTENISKSGSHLLDVINDVLDISRLELGNIELYYETVDIPGVIEEVRRV
LSSLSAEKNIRIEYKVEQGLKTIDVDRVKFKQILYNLLNNAIKFSSEDGKVNIKARSEGDMVEISVKDEGIGINEADYARVFL
PFVQIDESISRKHGGVGLGLALVKRFVELHGGQVWVEASPGKGSTFTFRIPKRP
>MA2555
NMSHELRTPLNSIIGFSDLLYEKIYGDLNEKQLKAVGNISRSGKHLLNLINDILDLSKVEAGKLELEYKEFELSSKLNSIKNL
LSPIADRKMIEVQIQVDESLNTIRADEARFAQIMYNLLDNAIKFSKENGLVEIDAKRKGDTVEITVKDYGIGIKVEDQSKLFK
PFSQIASFSSKKVQGTGLGLALVKQIVNLHGGYIWFNSRIGEGSTFAFTIPING
>MA1739
NISHELRTPLNSIIGFSDLLCEQIFGELNEKQLRYAGNISKSGKHLLSLINDILDLSKVEAGKMELDYTEFELAGKLNTIKNL
LAPIADRKSIQIEIEVDSRLTNLYADEAKFAQIMYNLVDNAIKFASENSPVNVGARMKGDKVEITVTDIGAGIKPEDQHKLFK
PFSQVDYFASKQHQGTGLGLYLVKQIVQMHRGYVWFRSVPGEGSTFAFAIPING
>MA3368
NMSHELRTPLNSIIGFSDMLYEQAYGELNRKQLRAIGNISSSGKHLLNLINGILDLSKIEANKMELNYREFDLATKLELIRNV
LYPVADKKNIDIEIDMDTELTKICADEEKFTRIMYNLVDNAIKFSYENSLVKIGARKNGNLVEITVTDAGIGIKAEDQHKLFK
PFSQVNSFPSKKFQGTGLGLSLVKQIVNLHGGYVWFRSEQGEGSTFAFAIPIAS
>MA2553
NMSHELRTPLNSIIGFSDLLYEKVYGELNLKQTKAVGNISNSGKHLLNLINEILDLSKVEAGSFELHYSTFWLAEVFAEVRDM
IFPFATSKGLKIELEIDSNLSRVYADKERILQVLSNLVTNAVKFSNENGCVKVKAVQMDGFLKITVADDGIGIAAADHEKLFK
PFSQIDSSFSKRYQGTGLGLALVKEIVQLHGGTVWFESEVGKGSVFGFSIPLPG
>MA4377
NMSHELRTPLNSIIGFSDILIERVFGELNEKQLKYVNNISGSGKHLLGLINDILDLSKVEAGKMDLHYSEFTVDSVFEEVKST
LFPLAQAKSLEINFVVGPDFGDIQADRSRLIQILYNLVSNAIKFTPEGGRVSVYCKKSGSRALFSVTDTGIGISSEDQKKLFQ
PFTQIDSSSARQYCGTGLGLALVKKIVNLHKGDIWVESELEKGSTFMFTIPLTK
>MA2348
NMSHELRTPLNAVIGFADILNEEGFGPLNKKQKRFVGNISTSGKHLLKLINEILDLSKIEAGKMEFKCTEFSVKEKFDEIKDI
LFPVFSKKKIRIEFEIGDGITTIYSDEGKFVQVLYNLVSNAIKFTEEGGFVKVSARKNGDMLQLSVKDTGIGITEEDLMKIFH
PFVQVDSFSTRQYEGTGLGLALVDQMVQLMGGEICVFSQPGIGSEFICMIPLKN
>MA0777
LKKAFYNLFDNARAHGDHVSEIDVSSHIVGESVVIEVKDNGIGVSPVMKELIFEKSVGRNTGLGLFLVRGILSITGMEITETG
IEGEGARFEIKVPPGN
>MA1957
TVSHELKTPLNSIIGFSDLLLEDSSGKLSERQARYINNISISGKHLLQFIDDILDLSKIEAGKTFLEPENFEFTKIFKDIEKV
FRPRVSGKKLSLNFEVDSGIKSFYADKMMFKQILYNLISNAVKFTPEEGSITVSAAKIGNMVRICVKDTGIGISREDMDSFFQ
PFKQPDSFFKRRYERTGLGLFLVKRFVEMHGGNIQAESVPGEGSSFIIELPLKT
>MA2256
TMSHELRTPLTAIIGFSELMLGEATGEFDELNRKFLGHISNSGKHLLSLINSVIDLSRIEAGKMDLEPDFFSLYDIFADTKSI
SSPLALKKNISMDFNVESDFLIYADRTRFKQIMYNLVSNAIKFTPAGGSVEVVGRRSENRIRVTVSDTGIGISQDEIKHLFKP
FKQINFALSREYESTGLGLVLSKNFVEMHGGRIWVESEPGKGSTFTFEVPVEI
>MA2013
NMSHEIRTPMNAVLGMLEMLLETSLTDEQREYLQLAHASAESLLSIIDDVLDFSKIEQNKLELEQISFELESLISHIINLLSG
KAGSKGLKLAFHIEKDLPSTFIGDPVRLKQVLFNILGNAIKFTKKGEIALSVEVYMPSERNSGLSNSDFPEEVALLFKVKDTG
IGIPPEKLSQIFDPFIQADASVTREYGGTGLGLAISSQLVELMEGRIWVESEVGKGCTFYFTVRVKR
>MA1270
VSSHDLQEPLRMIASYLQLLQRRYQGELDERADKYIYFAVDGASRMQSLINDLLEFSRVTTKAREFEPTDCESILNYVLSDLE
VSIKENEATVSYDSLPEIMADGTQLTQVFQNLISNAIKFRSKEAPKIHVSAEKEDDKWRFSVQDNGIGINPKYSEKIFEVFKR
LHKREEYPGTGIGLSICKKIIERHGGDIWVKSEPGRGSTFYFTLPASF
>RdmS
EDQQQKAIDTVINSSERLKHMVDSLLYLSLEQAGKIEYSFGEVEIKKILSDVYLNLVLLIDEKELKVEKELPASLPPIRGDKQ
KLTDLFTTLMGNSIKFTPHGGTLEVKAEEEEETIHITLKDSGTGIQKRLIPHLFHRIYQVDDSLTRRYQGLESGFYICKNIVN
AHEGEIWVESEEGSGTTMHVRLPKKK
>MsmS

EFVEEMMFPEKAEYGEIMDYETLYAIDSQQQKAVNTFIHYSEKLRRLVDSLLYQSLEKAGKIDYSFEETQLKDVLSDAFLNNV
FLIGEKALEVKKEVSASLSEIKGDREKLTALFTALIDHAIKFTPQGGKLALEVKEEAGNVHIVIADSGKGISKELIPYLFDRL
YQVNDSITRRYQGLESGLYICKNIVDAHKGEIWFESEEGLGNLMHVKLPK
>MA0203
EIHHRIKNNLQVISSLLDLQAEKFNSREDIKDSEVLEAFRESQDRVISMALIHEELYKGGEFDTLDFSSYIEELTENLFLTYR
LGNTDISLNMDLEENIFFDMDTAVPLGMIINELVSNSFKYAFQGRNRGEIRIKFRREENGKYINSGVKSKNGGCKSNNFTLTV
SDNGVGIPENFDIEDLDSLGFQLITSLVDQLDGKLELKRNNGTEFTVRFTVRD
>MA0490
EIHHRIKNNLQVISSLLSLEAEKFSDEKMLESFRESQNRVASMALIHEELYKGNELDTLDFAAYLQKLTADLFDSYNLGDSGI
SLKLDLEKIHLDMDIAIPLGIIVNELVSNSLKHAFSAGKAGEIHISFCKKESFAANDDIPGPCPFCTGKNNLHYILTVADNGK
GIPEEIKFPNTDSLGLQLVNLLVEQIDGYIELKSDSGTKFTIWF
>MA0551
EIHHRIKNNLQVISSLLDLQAEKFQNKEVLEAFRESQNRVTSMSLIHEELYKGGENNTLNFSTYLQKLAENLFQTYSLKSKKV
LLCMDLEENTLFDMDIAVPLGIIVNELVSNSLKHAFTEEEEGEIRIKLCREEKGNEMHRSLFSLSISDNGKGIPESTKLESIE
SLGLQLVSILVDQLDGKIELKRTHGTEFRITFNVTE
>MA0552
EIHHRIKNNLQVISSLLDLQAEKFNNREDIKDSEILEAFRESQDRVISMALIHEELYKGGGFDTLNFSSYIEELAENLFQTYS
LGKADISLKMDLEERIFFDMDIAVPLGIIVNELVSNSLKHAFTEGEEGEIRIRLCREKKNNELDKSLFSLTISDNGNGIPENV
ELKSFESLGLQLVSILVDQLDGKIELKQEQGTKFRITFKVAE
>MA0619
EIHHRIKNNLQVISSLLSLEAEKFSDERTLEAFRESQNRVVSMALIHEELYEGKGMDTIDFAVYLRKLTLDLFNSYTVETRKV
RLNLDLEQVYLGMDTAIPLGIIVNELVSNSLKHAFPSGGEDEIRINLRRMEDSTSKPERVPGCQNGKVFHYMLTVTDNGRGFP
EEINFQNSESLGLQLVNILVEQIDGCIELKRDRGSEFAIFFDNPG
>MA0620
EIHHRIKNNLQVISSLLSLQAEKFEDREVIEAFRESQNRVASIAMIHEELHGGENLDSLDFADYLQKLTADLFDSYRVGKEGV
SLKLDLENVYLGMDTAIPLGIIVNELVSNSLKHAFSDKNEGEISINLHSNDASHSLVELSCSEPEFLENDDFHYTLTVADNGR
GIPEKMDFRNTDSLGLQLITILVEQIGGCIELNRDYGTEFVINFGNSE
>MA0758
EIHHRIKNNLQVISSLLDLQAEKFRDKDVLEAFRESQSRVLSMSLIHEELYKGGETDTLDFSTYLEKLAENLFRTYSFRSKNI
CLSMNLEENAFFNMDIAVPLGIIVNELVSNSLKHAFTEKEGDIRIRLCREESDCEMSKSLFSLTISDNGKGIPENVELESVES
LGLQLVNILVDQLDGNIKLKHDQGTECRIEFKVME
>MA0759
EIHHRIKNNLQVISSLLDLQAEKFRDKEVLEAFRESQSRVLSMSLIHEELYKGEGTDTLDFPTYLQKLAENLFQTYSFRSKNI
RLYMDLEENAFFNMDIAVPLGIIVNELVSNSLKHAFTENKEGEIRIKLCREEKNNEMQESIFSLTISDDGKGIPENIELENIE
SLGLQLVNILVDQLEGNIELKRDQGMESRIEFKVMER
>MA0970
EIHHRIKNNLQVISSLLDLQAEKFRGKKNIEDSKILEAFKESQDRVISMALIHEELHKSGEIDTLNFSAYIHELSGNLFLSYR
LGNDGISLDMDIEEDIFFDMDTSVPLGMIVNELVSNSLKHAFSDRDKGEIRIKLHRKKNRESDIEDCCIAFILSVSDNGIGIP
KDLEIEDIESLGLQLVTTLIDQLDGELELKRDDGTEFIVRFSITE
>MA1274
EIHHRIKNNLQVISSLLDLQAEKFRSREHVEDSEVLNAFKESQERVISIALIHEELHEGKGTDTLNFSPYLQRLVKNLFQIYN
LGNVDISLSMDIEENVFFDMDTAVPLGLIVNELVSNSLKYAFKGRDKGVIRIKLSREGNGEIMSNREESKKEGHEDTNFVLTV
SDNGVSIPEDFNLENSDTLGIQLVTTLVDQLDGRLEMKRGSGIEFIVRFPITE
>MA1322
EIHHRIKNNLQVISSLLDLQAEKFGNKKYIMNSEVMDAFRESQDRVISMALIHEELHKSEGLDTLNFSPYIEELAENLFQTYR
LGNSNICLNMDLEENIFLNMDTAVPLGIIINELVSNSFKHAFTEDEEGEIRIKLHREENGEHKKERNKSTDFVLTVSDNGAGI
PENLDIEDLGSLGLQLVTSLVDQLDGELELKRENGIEFTIRFTVTE
>MA1470
EIHHRIKNNLQVISSLLELQAEKFDNLEVLEAFRESQNRVATMAIIHEELYRSRNNETLDFSAYLQKLTADLFHSYLVRKGDV
GMQLNIEEIFLGMDTAVPLGIIINELVSNSLKHAFPSGRKGEIYISLCRTDENNENKSISNIINNMDAKSPVDKNIQYMLVIS
DNGLGFPENVDFTNTSSLGLQLVNILVEQLEGAIELENDSGTKFKIWFKEPC
>MA1627
EIHHRIKNNLQVISSLLDLQADKFDNPKVIEAFRESQNRVISMALIHEELYKGEGNDTLNFSTYIKELAGNLFQTYSLTSKNI
CLCMDMEKNVLLNMDTAVPLGIIVNELVSNSLKHAFSGKEGGEIRIKLRRKGNGSRKKEGDKATSFILIVSDNGIGIPENLNI
QDVDSLGMQLINTLVDQLDGKLELKRGNGTEFTIKFAVAE
>MA1628
EIHHRIKNNLQIISSLLDLQAEMFKGRSNIRDSEVLKAFAVSMDRVLSIALIHEELYKGKNIDVLNFSEYIKKLADNLLITYR
LETDVNLNLDLEENLFLNMDAAIPLGIIINELVSNSLKYAFPDRDKGEIRIKLRREEKGECKINGCKSADFVLTVSDDGIGIP
ENLDIKDLDSLGLQLVISLVDQLDGELKLKRGNGTEFTIKFAVTE
>MA1630
EIHHRIKNNLQIISSLLDLQAEKFEDKNVTEAFREGQNRVISMSLIHEELYKGEGTDTLDFSVYLKKLAENLFQTYNLSSKNI
NLSMDLEKDTFLDMDTAVPLGIIVNELVSNSLKHAFIEEGKVRINLCREERNYDTNESLFSLTISDNGKGMPEDLELESAESL
GLQLVNILVDQLDGELELKRAQGTEFTIRFKVVE
>MA1645
EIHHRIKNNLQVISSLLDLQAEQFKNRENIKDSEVLEAFRESQDRVISMALIHEELYKGGGFETLNFSPYIKELVENLFQTYR
LGDIDISLNIDLEENVFFDMDTAVPLGMIVNELVSNSFKHAFIGRDKGEIRIELYREESAEFESENRKSTNFILTVSDNGVGI
PDNLDIEDLGSLGMQLVVSLIDQMNGELELKRNNGTEFTMKFTVTE
>MA1646

ELHHRIKNNLQVISSLLDLQADLFKGKKTITDSEVLKAFNESIDRVLSIALVHEELYKGKNIDLLNFSQYIKELANNLLLTYS
LKTDVSLNFDLEENFFLDMDTAIPLGMIINELVSNSFKYAFPERDKGEIRIKLRREEKGKCKINGCKYADFVLTVSDDGTGIP
ENLNVKDLNSLGFQLVTSLVDQLGGEFELKRNNGTEFTMGFSVIE
>MA1704
EINHRIKNNLQVISSLLSFEAEKSTDPEILEAFRETQNRIASMSLIHQELCIGETYTIDLADYLRKLTAGIFSSYLVGNERIN
LRLDLEQVYMETDTAVPLGIIVNELVSNALKHAFLPGKEGEIRVNLSRMKNCEKEFKNSRSSGIVPGYSNGKNLQFILTIEDN
GRGIPELGDHQNKDSLGLQLVNIFVEQIGGSIERKKDKGTKFNIRFNKLE
>MA1844
EIHHRIKNNLQVISSLLELQADKFKDREVIEAFRESQNRVASMAIIHEELYRAGDIETLDFSAYLRKLTSDLLSSYTVRKEDV
KLKLEAEDTFLGMDTAIPLGIIINELVSNALKYAFPAGRRGEIRIKLCRKEINENKNIDEIISNNYRGSSIKNSYLYSLVVSD
NGLGFPENVDFKNTDSLGLQLVNILVEQLEGTIEMEKNGGTTFRISFTETE
>MA1878
EIHHRIKNNLQVISSLLDLQIDIFSNREICKTPEVIEAFRESQNRVVSVALIHEELYKSKGMDSLDFAAYLQKLTKNFLKSYN
IDADDINLKLDFEQVYLGMDTAVPLGIIVNELFSNSLKHAFPNKREREIKITLQKEENVYKKASFHYMLTVTDNGKGIPEEID
IQTADSLGLQLVNILVEQIDGCIELKRNKGTEFTIWFNNIE
>MA1991
EIHHRIKNNLQVISSLLSLQAEKFRDQEVLEAFRESQDRIISMTLIHEELYKGGGTDTLNFSKYIQKLTENLFRIYGLKSKNI
CLFMDLEENAFFNMDTAVPLGIIVNELVSNSLKYAFIDKQEGEIRICLCREEKNDGMNRSLFSLIISDNGTGIPENVELENVE
SLGLQLVNILIDQLDGEIELKRDHGTEFRINFEVAE
>MA2082
EIHHRIKNNLQVISSLLDLQAEKFRDKEVLEAFRESQNRVVSMSLIHEELYKGEGTDALDFSAYLRKLSEKLFQTYSLSSKNI
RMYMNLEENTSFNMDIAVPLGIIVNELVSNAFKHAFPEKVGEIRIQLQKEKMCNEVNRSLFSLIISDNGIGIPEGVELASFES
LGMKLVNTLVDQLGGKIEIIRAHGTEFRITFNVTE
>MA2266
EIHHRIKNNLQVISSLLSLQAEYFSDPKVKESFKDSQNRVISMSLIHEELYKTRETADIETFDFKVYIQKLATELFKSYLVGS
EDIRLKLDVESAFLGMDTGIPLGIIVNELVSNSLKHAFPEGRSGEIQIKLHRTGSSQKKGCNKHPGNCEISEFLLTVSDNGIG
FPEDLDLKHTSSLGLQLVNILVEQIEGSIELERGKGTEFRLKFREQE
>MA2732
EIHHRIKNNLQVISSLLDLECDSLLSGTPDHKKIAEAFRESHNRIISMSVIHEELYNSRDMETINFASYLKKLTDDLFKSYKV
GNSDIKLYLDVEDFFFEMDNAIPLGIIVNELVSNSLKYAFPDGRNGEIHIELQALDDKKSSNTTADFSMQNNVGPSSYFRLTV
GDNGIGFPGSFDFKNISSLGLQLVNTLVDQIGGSIEIGNGPGTKYNILFKDS
>MA2757
EIHHRIKNNLQVISSLLDLQAEQFKNRECIKNSEVLEAFRESQARVISMALIHEELYKGDGLEMLNFSPYIEELAKSLFHTYR
IGNSDIRLKLDLEQNISFDMDTAVPLGIIVNELVSNSLKHAFPDGGTGEITIKLHREENGEQINNPNKNCSVNFILSVSDNGV
GIAENLNIEDLDSLGLQLVTTLVEQLNAELELKRNNGTEFILKFIVTE
>MA2784
EIHHRIKNNLQVISSLLDLQAEKFKDREDIKDSEVLEAFRESQDRVISMALIHEELHRNEGLDKLNFSQYIKELADNLFLTYK
LGNDGTRFNKDIEENIFFDMDTSVPLGIIINELISNSLKYAFQGRNHGEIQVKLYREEDREQDIEDLNSTAYVLSVSDDGVGI
LKDLDIEELDSLGLQLVTSLVKQLNGELELERNNGTEFIIRFTVTE
>MA3346
EIHHRIKNNLQVISSLLDLQAENFSKHEVCKTPKVVEAFKESQDRVISIALIHEELHENGETDTLDFSPYLEKLVDALFQTYR
LGNARITLKKELEKNIFFDMDVAVPLGLIVNELVSNSLKHAFSDRDSGEIDIKLCREISPEQETDLCSENETKSRRETGFILT
VSDDGTGISDAVDLENSDSLGLQLVKILVDQLEGEMEVKREKGTEFTIRISVAE
>MA3370
EIHHRIKNNLQVISSLLDLQAGKFNNKEHIRDSEVLEAFKESQDRVTSIALIHEELHEEEGKTTDTLNFPIYLQRLVKNLFRT
YTLGNIDISLNLDLKENIFFDMDTAVPLGIIVNELVSNSLKHAFSGRNKGIIQIKLFSEEAGNEPNSKRKLSMEKLIKEKLAE
EELAEEELAKEELAKERIPGKSTGYTLIVSDDGIGIPEEIDIKNPETLGLQLVNILVDQLDGKIKLEREHGTEFIINFSVEE
>MA3481
EIHHRIKNNLQVISSMLSLQAEKFSDEETLEAFRESQNRVTSMALIHEELYEGKDMETLDFAVYLRRLIGDLLSSYTVGNREI
DLKLDLKQIYLGMDTAVPLGIVVNELVSNSLKHAFPAGKRGEIRISLSRSENYEKRHENGNFEVERYIETGSRSIEPDEMGIE
KSEEGIVRCSGSKRGIEKSEKDPVFMLIVADNGNGFPQNIDFRNTDSLGLQLVSILVEQINGSIELNRDEGTEFRILFGKVG
>MA3543
EIHHRIKNNLQVISSLLDLQAEKFNKREGIKDSEVMEAFRESQDRVISMALIHEELHKSGGLDKLDFSSYIKELADNLFLTYR
LGTIDVSLNMDLEKNIFFDMDTAVPLGMIVNELVSNSLKHAFLGRDRGEIRIKLRREGNRECRNSIGCVESNSKDCESPSFTM
IVSDNGVGIPKDLNIEELDSLGFQLVISLVEQLDGELELKRDKGTEFIMKFTVTE
>MA4026
EIHHRIKNNLQVISSLLDLQAEKFSHREAVPTLEILEAFKESQNRVISMSLIHEELYKGEGTDTLNFSVYLRKLAENLFQTYS
LCSKNIRLYMDLEENTFFNIDVAVPLGIIVNELVSNSLKHAFAENKEGEIQIKLCREESDCEMSKSLFSLIISDNGKGIPENV
ELGSVDSLGLQLVSILVNQVDGKIELKKAQGTEFRIIFSITE
>MA1267
EIHHRIKNNLQVISSLLDLQAEKFEDPTIRQAFRESQNRVISMALIHEELYESGEIGTLNFAAYMQKLVENIFECYNIGDHKA
HLHLEIEEKTFLDMDTAVPLGIIVNELFSNSLKHAFPERDEGIVKIRLCREENWECKSNSVENTGVENPGRDNTRGDNKSINL
ILTISDNGVGMPETVDMESPCTLGLQLVTILVDQLDGEIELKRDSGTEFIIKIAVKQ
>MA1463
EINHRIKNNLQIVSSLLDLQAEQFSDKKVIEAFRESENRIVSMSLIHEELYESGNLDILDFSSYIRKLIDDLCRSYSTESSHI
RIKLDVETVFLGVNLAVSLGIIINELFTNSLKYAFSPGKGEEISISLLREDTRGQDQRPEAISEKVFLTASEDSENFTLVFAD
NGKGFPEESQFQKHGIPWAAACKCSCEPDRGEHRP
>MA2890

EIHHRIKNNLQIVSSLLSLQADKFKDKDVIEAFRESENRVISMSIIHEELHKSEDTTNIDFAAYLRKLTSELLYSYKVGNEKV
RFFLDVDNVFLGIDTAIPLGIIINELFSNSLKYAFLKSAEGEIRISLYRQPEVLELCSTSGRGQNTGSVVHSSFTLVYSDNGG
RFPENIDFKNPETLGLQLVNALVEQLDGTIELEKGGETKFIIRFDDKGLPGKS
>MA0014
RISTEQLDKLMNLVGELVINRSRVKELTGESKSKDLELALSEFQKLTRELQEEVLEIRMVPLDHITNIFPRMIRNLAREQNKK
INLVIRGKEIKLDRAIMEEIGDPLVHLLRNAVDHGIELPEQRVELGKEETGTIMITASKQQNYVLVKIEDDGRGINAKEILQA
ALEKGFISRDEAEQLSEREAIQLIFAPGFTTASTVTDLSGRGVGMDVVKNRIEHLGGSVKVESKLGFCSRFELRLPITI
>MA3066
GSSHHFSESAASAKTPLETQRQETIRVRTSNLDNIMNLVGELVINKGRLLQISQEYNLPELEEATGALDKSISSLQDEVMLIR
MVKIERVFSKFPRMVRDLSRKFDKNIEFEIEGQDTELDRTILDEISDPLVHLVRNAVDHGIEYPEAREKAGKNEVGNIKLSAR
REKNNVIIEIEDDGKGIDVEVLKKKAVEKGIFSAFDVENLSEEEIRMIIFSLGFSTKESPTEISGRGVGMDAVKTAVEKLGGK
VRVYSKKGEFTRIRIDLPPTV

>MA0016
MARVMIVDDAEFMRMVIRDILLKHGHEVVAEVGDGEEAIQTYLEVKPDLVLMDIIMPDMDGKEALQKLLLIDPDAKVVMCSSL
GQQALITESMKIGAMGFIIKPFEPDGMLDVIKKIAEPN
>MA0018
MPEILIVEDNLLNLVIEADLLKSCGYDPKKAKNGFEALEVLSKVKVDLVLMDMELPKMHGLELLQRIKCNPETQGIRVVAVTG
HCDPESEQEFLKAGCHAVLSKPINFDLFGAQVKEFLTATNSPG
>MA1268
MKTKVAAKPIEILLVEDSEGDVGLIEEVFEEAKIRNNLHIVEDGEEAILFLRGEKQFSGISRPDIILLDLNLPKKDGREVLEE
IKEDDDLKNIPVVVLTTSKAEEDVLKSYNLHANAYVTKPVDFDQFIRVIKSIEDFWLEVVKLPSK
>MA1269
METWTAFKPVDILLVEDNKGDVGLIEEVFESSKVRNKLYVVEDGEEAVHFLLREGKFSDVPRPGIILLDLNLPKKDGREVLEE
IKEDDDLKNIPVVVLTTSRAEEDILESYKLHANAYVTKPVDFDQFIKVIKSIENFWLEVVNLT
>MA1366
KILIMGNGNNVHNNLQKVLEAENYNVVSASDNFSAIETVNEEKPDLVLLDTVYLETDGFEICRQLKDSPRYWWIPIMMLSERN
KTEDGIKAFDSGADDYITMPFNPLELKARVGMIL
>MA1468
MDKAKILVVEDQNIVALNLRNRLKNMGYIVPTIAISGEEAIRKTELTTPDLVLMDIMLKGDMDGIEAARIIKSRFSAPVIYLT
ACTDIGILERAKLTEPAGYISKPFKEKDLVSNIEVALQKNKLGKVTEE
>MA1469
MKKAKILVVEDQNIVALNIRNKLKNLGYTVPGTASTGEEAIRKAELTNADLVLMDIMLKGDMDGIEAAREIKARLKIPVLYLT
AYTDDETLERAKMTEPAGYISKPFKEEDLHSNIEMALHKHRTEKKEIENSDSDE
>MA2012
KVLIAEDEPISNLWLKNTLTRWGYEAISTRDGYEAWEVLNESDFPRVVILDWEMPKMKGIEVCEKIKKDPRLSSIYIIILITGR
DLTEDMEAGFKAGADDYLKKPFDNRKLKTKLDTAR
>MA2013-R1
NVLFAEDHPINQKLILGLLEKKGHKLTLVTSGKDALDALSRRDFDAVLMDIQMPGMDGLEATRRIRDPSSGVRRHNIPIIAFT
ARALKEDREKCFEAGMNYYISKPLKKEKLLNILEDIR
>MA2445
KVLIVDDKKENVELMEAYLAVEPYDVITAYGGKEAFQKVKEEKPDIILLDVMMPEVNGYEVCKILKGNPETQFIPVLMLTALS
ELEDRIRGIEVGADDFLTKPINRLELKTRVKSLL
>MA2861
MKVLLVDDDPVFLELSKTFLEVFHDINSDTVESARQALEKLDELSYDVVVSDYDMPYMDGISFLKTIRDKRINIPFILFTGVG
KEEIKSQAIENGVDSLIQKRGDPKAQYSELSKRIWQIVKNGSG
>MA3068
MAKVLIVDDTAFMRKLLKNILFGAGFDIAGEAENGKQAVEMYKGLKPDVVTMDVVMPEMTGIDALKQIKALDKDAKIVMCTAI
GQENIVKTAIKLGARGYIIKPFQAPKVIEEIKKVIGA
>MA4376
MKEILIVEDNPMNMELILDLLEFYGHRVTEAEDGIKALERLAEKKFDIILLDMQLPKMDGLEVLDRIKKNPATADIPVIAVTA
HAMKGSEEHFIEMGCVDYISKPIDIHRFRSLIDKYLG
E
>MA4377-R1
LVLVVDDDINSNELISVVLREAGYSTASLHNGKDVLEVAKKLKPDVITLDVLLPDTSGWNVLKQLKSDLDTTSIPVLIISVTD
NNELGVALGATYSFTKPVRRVELLDSLREIT
>MA4377-R2
KVLIIDDDENAVELLSSMIESEGFEIVKAYSGQAGLDKLFSEQQPDILILDLLMPEISGFEIISRLRDGEQTKDIPLIVCTAG
EFTEKNIEKLNGELKGHLISIMKKGTFGRKELINRIKQLA
>MA4671
MEMKSILVVEDSPVILELISFFLTSSGYESRETGDGFDALKIAEENRFDLILLDKQLPGFDGLEVLKKIKKIFEIRKTSVIAL
MAHAMQGDEDRFLKAGCNGYISKPIDIDRFKLILDTCTGGYQVL

References

Grant JR, Enns E, Marinier E, Mandal A, Herman EK, Chen CY, Graham M, Van Domselaar G, & Stothard P. (2023). Proksee: in-depth characterization and visualization of bacterial genomes. *Nucleic Acids Research,* 51(W1), W484-w492.

Grozdanov L, Raasch C, Schulze J, Sonnenborn U, Gottschalk G, Hacker J, & Dobrindt U. (2004). Analysis of the genome structure of the nonpathogenic probiotic *Escherichia coli* strain Nissle 1917. *Journal of Bacteriology,* 186(16), 5432-5441.

Guss AM, Rother M, Zhang JK, Kulkarni G, & Metcalf WW. (2008). New methods for tightly regulated gene expression and highly efficient chromosomal integration of cloned genes for *Methanosarcina* species. *Archaea,* 2(3), 193-203.

Hanahan D. (1983). Studies on transformation of *Escherichia coli* with plasmids. *Journal of Molecular Biology,* 166(4), 557-580.

Kollmann R, & Altendorf K. (1993). ATP-driven potassium transport in right-side-out membrane vesicles via the Kdp system of *Escherichia coli*. *Biochimica et Biophysica Acta - Bioenergetics,* 1143(1), 62-66.

Kwiatkowski K. (2013). Integration of pyrrolysine and characterization of a sensorkinase from *Methanosarcina acetivorans*. Master thesis. *Ruhr-Universität Bochum*.

Miroux B, & Walker JE. (1996). Over-production of proteins in *Escherichia coli*: mutant hosts that allow synthesis of some membrane proteins and globular proteins at high levels. *Journal of Molecular Biology,* 260(3), 289-298.

Sexauer A. (2021). Characterization of a bacterial-like signal transduction phosphorelay in *Methanosarcina acetivorans*. doctoral thesis. *Technische Universität Kaiserslautern*.

Studier FW, & Moffatt BA. (1986). Use of bacteriophage T7 RNA polymerase to direct selective high-level expression of cloned genes. *Journal of Molecular Biology,* 189(1), 113-130.
